# Editorial: Cross Talk between Lymph Node Lymphatic Endothelial Cells and T-Cells during Inflammation and Cancer

**DOI:** 10.3389/fimmu.2017.01421

**Published:** 2017-11-20

**Authors:** Sonia Elhadad, Silvia Della Bella

**Affiliations:** ^1^Department of Medicine, Weill Cornell Medicine, New York, NY, United States; ^2^Department of Medical Biotechnologies and Translational Medicine, University of Milan, Milan, Italy; ^3^Laboratory of Clinical and Experimental Immunology, Humanitas Clinical and Research Center, Rozzano, Italy

**Keywords:** T cells, lymphatic vessels, lymphatic endothelial cells, inflammation, cancer, antigen presenting cells, atypical chemokine receptors, microRNAs

**Editorial on the Research Topic**

**Cross Talk between Lymph Node Lymphatic Endothelial Cells and T-Cells during Inflammation and Cancer**

A successful adaptive T cell immune response depends on the encounter of T cells and antigen-presenting cells (APCs). Therefore, T cells constantly patrol the body, recirculating between the blood and the lymph nodes, looking for antigens drained from peripheral tissues. In the lymph node, lymphocytes recognize antigens upon contact with APCs, proliferate to expand few clonally relevant lymphocytes, differentiate into effector T cells, then exit the LNs and migrate to peripheral tissues to ensure immune protection. All these processes involve cellular interactions and migration. Lymph nodes are strategically distributed throughout the body, at the junction of the blood vascular and lymphatic systems. T cells spend several hours in a lymph node sampling the microenvironment, leaving then the lymph node *via* efferent lymphatic vessels (LVs). Traditionally considered as passive conduits, LVs appeared to be active players in the modulation of the immune response. The lymphatic system serves as the primary route for the metastasis of many cancers, and the extent of lymphangiogenesis is an important indicator in tumor progression. The promiscuity of immune cells and LVs suggests that immune cells modulate this biological process during inflammation and cancer. Those, cross talks between LVs and the immune system can be used for therapeutic strategies for cancer and other pathologies.

This Research Topic brings together eight articles that provide insights into the various biological functions of the LECs, the ways they regulate the immune responses, and the therapeutic strategies that can be developed.

In their mini review, Yee et al. focused on the role of microRNAs (miRNAs) as regulators of LECs’ function. The authors discuss the role of miR-31 and mi-R181a that targets PROX1, the transcription factor controlling the upregulation of LECs’ markers. As a result, LECs’ specific genes expression is repressed, therefore miR-31 and mi-R181a control LECs’ differentiation and plasticity. Moreover, elevated levels of these miRNAs are found during inflammation suggesting a possible role of these miRNAs in inflammatory lymphangiogenesis. The authors suggest that they can be used as new therapeutic tools in inflammation and cancer.

Chemokines and their receptors are key factors in LVs’ function. In their mini review, Bonavita et al. discuss the role of atypical chemokine receptors (ACKRs), and in particular the role of ACKR2 in lymphatic biology. The authors reported data showing the essential role of ACKR2 expressed by LECs, in regulating chemokine concentration and leukocyte migration, promoting therefore resolution of inflammatory responses in infection, allergy, and cancer. Finally, they speculate that ACKR2 could be considered as a potential therapeutic target to attenuate inflammation during psoriasis and lung infection, or by influencing cancer cell dissemination to metastatic tissues.

Lymphatic vessels are key players in the cellular migration that accompanies lymphocytes patrolling during homeostasis and inflammation. In their review, Hunter et al. discuss in great details T cell migration within and between peripheral tissues and secondary lymphoid organs. While T cell migration within the lymph node occurs in a one manner where recirculating lymphocytes exit through efferent LVs to return to the blood circulation, in peripheral tissues T cells exit through afferent lymphatics to migrate to draining lymph node, before joining the blood circulation. The authors discuss further the relevance of T cell migration through afferent LVs in immune surveillance and resolution of local inflammation, and the use of this migration process for the development of immunomodulatory therapies.

Number of publications reported the function of LECs as APCs. In their review, Humbert et al. discussed how this property shapes the immune response. LECs express a large range of peripheral tissue-restricted antigens (PTAs) and present class I restricted PTA-derived antigens to CD8^+^ T cells, leading to the deletional tolerance of self reactive CD8^+^ T cells. The authors discuss further the contribution of LECs as regulators of peripheral T cell responses in autoimmunity and cancer.

While these papers cover the regulation of the peripheral immune response by LECs through different biological processes, LECs’ function is itself regulated by T cells during inflammation and cancer. We have reported that temporal inflammatory lymph node lymphangiogenesis is regulated by a mixed Th1/Th2/Th17 response (1). Yeo and Angeli discuss further the cross talks between LECs and T cells and their implications in cancer, and the use of lymph node LECs as a potential therapeutic target in addition to immunotherapy strategies for cancer progression and metastasis.

Tumor vasculature plays a crucial role in shaping the tumor microenvironment and contributes to cancer immune evasion. In their review, Hendry et al. described the mechanical and molecular mechanisms underlying tumor-promoting properties of tumor vasculature. They also explained the design of combined antiangiogenic and immunotherapeutic treatments and summarized the drug combinations explored in preclinical and clinical settings. Promising results have been reported for antiangiogenic therapy combined with immune checkpoint inhibitors in different types of cancer, suggesting that each treatment may potentiate the effect of the other. We suggest that endothelial colony-forming cells isolated and cultured from blood may represent a tool for studying the endothelial compartment in cancer patients (2, 3), and assessing the impact of combined treatments during patient follow-up.

Dieterich et al. delineated the differential role played by lymphatic and blood vascular vessels in the tumor microenvironment, highlighting a role for LVs in promoting cancer immune tolerance. By using two different *in vivo* models, the authors showed that, during cancer development, tumor-associated LVs—but not blood vessels—upregulate the checkpoint inhibitor PD-L1. Notably, this effect is dependent on IFNγ production by tumor stromal cells. T cell interactions with tumor-associated LVs, and T cell inhibition upon contact with PD-L1-expressing LECs, suggest that characteristics ascribed to LECs in secondary lymphoid organs are shared by LECs present in the tumor microenvironment. As reported in Lukacs-Kornek’s review (Lukacs-Kornek), LECs’ functions are shared by liver LECs. Our knowledge on liver LECs is limited to the notion that they are increased in chronic liver diseases and cancer. Availability of markers for LEC identification will clarify the contribution of these cells to liver disease pathogenesis.

## Author Contributions

SE organized the Research Topic; wrote and edited the Editorial. SDB helped in Research Topic management and contributed to writing the Editorial.

## Conflict of Interest Statement

The authors declare that the research was conducted in the absence of any commercial or financial relationships that could be construed as a potential conflict of interest.
